# Integrative linkage and recombination analysis of 25 X-STRs across 7 linkage groups using pedigree-based and SNP-based strategies

**DOI:** 10.3389/fgene.2025.1727583

**Published:** 2025-12-18

**Authors:** Jinglei Qian, Xiaoqin Qian, Qiqi Ji, Zhimin Li, Chengchen Shao, Hongmei Xu, Fan Yang, Jianhui Xie

**Affiliations:** 1 Department of Forensic Medicine, School of Basic Medical Sciences, Fudan University, Shanghai, China; 2 West China School of Basic Medical Sciences and Forensic Medicine, Sichuan University, Chengdu, China; 3 Shanghai Key Laboratory of Crime Scene Evidence, Shanghai Research Institute of Criminal Science and Technology, Shanghai, China

**Keywords:** genetic marker, kinship analysis, linkage groups, recombination, SNP, X-chromosomal STRs

## Abstract

**Introduction:**

X-chromosomal short tandem repeats (X-STRs) are valuable genetic markers in forensic science for resolving complex kinship scenarios. However, the linkage relationship and recombination of X-STRs remain poorly characterized.

**Methods:**

Based on high-density SNP data with relatively low mutation rates, we developed a pedigree-based method to analyze the recombination relationships between these X-STR linkage groups (LGs). We used X-STRs data from 66 two-generation families and X-SNPs data from 602 X-chromosomal SNP trios from the 1000 Genomes Project. We investigated the linkage relationships among 25 X-STR loci, grouped into seven LGs, including three identified regions (Xp21.1, Xq21.31, Xq23) and four Argus X-12 (Xp22.2, Xq12, Xq26, Xq28), provided broader coverage linkage groups of X-STR.

**Results:**

We found strong intra-group linkage (Maximum logarithm of odds (MLOD) > 5) and near independence between groups (MLOD <1). Estimated recombination rates of X-STR data ranged from 0.0000 to 0.0487 within LGs, and from 0.1561 to 0.4133 between LGs, while the recombination fraction between the 7 linkage groups occurred in approximately 50% of informative meioses. LD decay analysis showed that *R* ^2^ dropped to 0.1 at a distance of approximately 3.7 kb, supporting the feasibility of using SNP-derived LD signals to infer STR recombination patterns at fine scale.

**Discussion:**

The family-based methods with X-SNP provide a more robust framework for evaluating X-STR linkage with the advantage of a relatively low mutation rate, high density and phased, particularly for newly developed loci lacking extensive haplotype databases.

**Conclusion:**

These findings contribute to a more precise understanding of X-STR inheritance and enhance their reliability in forensic kinship analysis.

## 1 Introduction

Short tandem repeats (STRs) are widely used in forensic kinship analysis. When autosomal STRs do not provide sufficient discriminatory power for relationship inference, X-chromosomal, Y-chromosomal, and mitochondrial markers can serve as complementary tools (Gomes et al., 2020). Among them, X-chromosomal STRs (X-STRs) exhibit a distinct inheritance pattern: males inherit a single maternal X chromosome (excluding pseudoautosomal regions), effectively representing a haplotype, while females inherit one X chromosome from each parent, enabling meiotic recombination between homologous X chromosomes. This unique genetic trait makes X-STRs particularly powerful in certain specific kinship scenarios, such as in cases of missing samples (one or both parents missing), patrilineal half sibling identification, and incest cases, where traditional A-STRs or Y-STRs may yield uncertain or statistically weak results. These highlights the significant practical value of X-STR in forensic cases (Gomes et al., 2020; Shrivastava et al., 2014).

Currently, several commercial X-STR amplification systems have been released. Previous reports showed that Argus X-12 Kit adopted X-STR markers from four well-established linkage groups located in Xp22.2, Xq12, Xq26, and Xq28 regions (Nothnagel et al., 2012; Bini et al., 2019). However, the genetic information yielded by these X-STR markers with only 4 linkage groups may not be sufficiently informative for the accurate inference of specific kinship relationships, especially in scenarios involving mutational or recombination events within a linkage group (Hering et al., 2015). Expanding the number of well-characterized, independently inherited linkage groups would improve the reliability of forensic inference. Other commercial kits, such as AGCU X19 STR kit (Zhang et al., 2016), Goldeneye 17X (Tao et al., 2020), and Microreader 19X ID System kit (Xiao et al., 2021), include additional X-STR markers located outside these four regions. Given that all X-STRs are located on the same chromosome, understanding their linkage status and recombination behavior is crucial for the forensic application (Tillmar et al., 2017). The linkage relationship of new developed X-STRs were usually investigated with the LD analysis with population data and a small part of the pedigree recombination analysis with recombination rate (Rc). However, in some cases, distinguishing intra-group recombination from *de novo* mutations remains challenging (Yang et al., 2021). The methods have shortcomings, such as requiring a large amount of family data and difficulty in determining parental genotypes. While the physical distance between two adjacent X-STRs in a linked group is very short and low recombination rates were observed between them, only a small subset of locus pairs exhibited significant LD in the population (Sufian et al., 2017; Zhang et al., 2016; Zhang et al., 2012; Yang et al., 2019; Yang et al., 2017). For instance, DXS10074 and DXS10075 are only 0.02 Mb apart, yet they were found to be in linkage equilibrium in earlier reports (Zhang et al., 2016; Yang et al., 2019). For highly polymorphic STRs, detecting significant LD may be challenging and require a relatively large sample size. The observation that certain X-STRs are weak against LD may reflect a lack of strong associations in the population due to these evolutionary factors, or simply the limited ability of population level LD detection to capture weak associations with STRs (O'Connor et al., 2011). This discrepancy may also be due to the effects of long-term evolutionary factors, such as historical recombination, mutation accumulation, or genetic drift, which could disrupt population-level LD even between closely linked markers. In general, the linkage relationship of these additional markers is not yet clear. This uncertainty can lead to errors in likelihood ratio (LR) calculations and affect the interpretation of kinship results (Tillmar et al., 2017).

Single Nucleotide Polymorphism (SNP) derived from Whole Genome Sequencing (WGS) have been applied in genetic genealogy studies (Morimoto et al., 2016). However, issues such as cost, accuracy, and lack of standardization still limit their broader application in forensic medicine (Kling et al., 2021). Therefore, X-STRs remain the conventional choice in forensic genetic analysis. On the other hand, SNP has the advantage of relatively low mutation rate and high density. We hypothesized that integrating phased SNP data from large public repositories can overcome the limitations of traditional family studies for recombination mapping. The high-depth 1kGP data provides a clear set of phased data, which directly solves the problem of undetermined genotypes that may affect recombination rate estimation in traditional X-STR studies, thereby improving the reliability of SNP analysis.

To address the limitations of X-STR recombination research and enhance analytical resolution, we incorporated phased SNP data from 602 trios in the 1000 Genomes Project (1kGP) (Byrska-Bishop et al., 2022) and extracted the phased X-SNP haplotypes of offspring and parents in regions flanking the target X-STR loci. Compared to the Hapmap data (Phillips et al., 2012) which used undetermined phase data, we directly identified exchange events to estimate the recombination rates between X-STRs. This approach provides a novel strategy to investigate X-STR recombination using publicly available SNP pedigree data. In our previous reports, three additional linkage groups on the region of Xp21.1, Xq21.31, and Xq23 were identified based on marker discrimination power and physical positioning on the X chromosome (Yang et al., 2021). In this study, we incorporated these three linkage groups with the four previously established ones (Nothnagel et al., 2012; Bini et al., 2019) bringing the total to seven. We systematically analyzed linkage relationship among 25 X-STRs within these seven groups using both family-based X-STR data and the SNP-based approach with 1kGP trios (Byrska-Bishop et al., 2022).

## 2 Materials and methods

### 2.1 DNA sample preparation

Blood samples were collected from 66 two-generation families (Table 1) from China with informed consent. The Genomic DNA was extracted from FTA cards using the ReadyAmp Genomic DNA Purification System (Promega, United States) and the concentration was determined using NanoDrop-1000 spectrophotometry (ThermoFisher, United States). The 2800M (Promega, United States) were used as control DNA.

**TABLE 1:** The information of families investigated in this study.

Father	Mother	Daughter	Son	The number of families
1	1	2	0	22
0	1	0	2	21
1	1	1	1	20
0	1	0	3	1
1	1	1	2	1
1	1	2	1	1

### 2.2 Data collection from 1000 genomes project

High-coverage whole-genome sequencing of the expanded 1000 Genomes Project (1kGP) cohort including 602 trios was used for the linkage and recombination research based on the location of the X-STR loci (Byrska-Bishop et al., 2022). To determine the recombination of two junction X-STRs from adjacent clusters, SNPs with minor allele frequency of ≥0.2 from 602 pedigrees in the phased 1kGP cohort were extracted (if the child is a male, the genotype data of the father is not needed) and the phased data was extracted by the SNP data was extracted and filtered from the VCF files by the ID of the trios with VCFtools (Danecek et al., 2011).

### 2.3 STR locus selection and primer design

**FIGURE 1:**
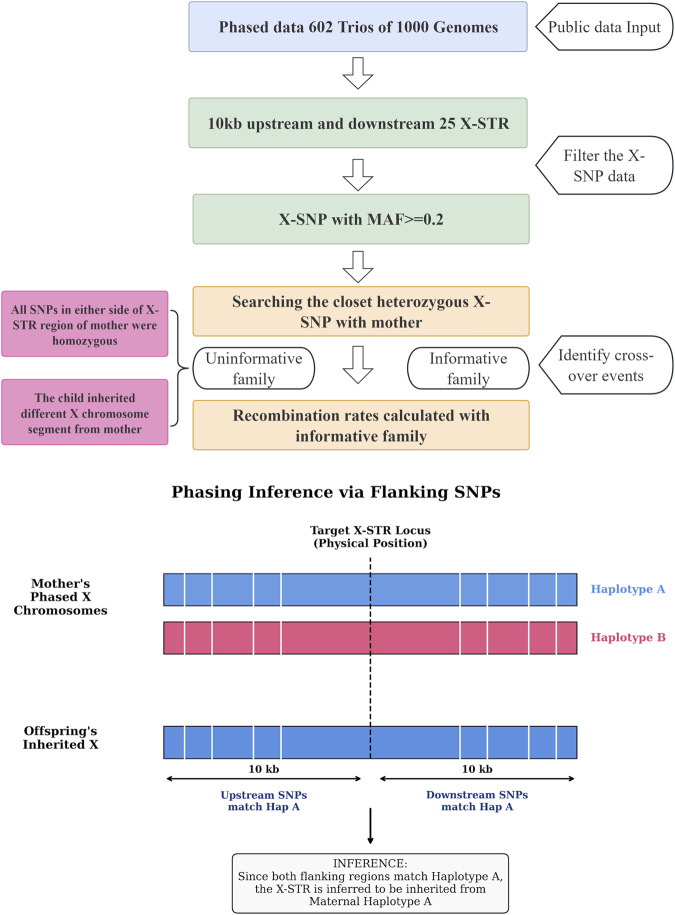
The workflow of recombination rates of linkage groups of X-STR with phased X-SNP data of 1000 Genomes Project.

Ten X-STR from three linkage groups in the region of Xp21.1, Xq21.31 and Xq23 in our previous study (Yang et al., 2021) were adopted based on the physical location in the human genome and the polymorphism in the Chinese Han population (Figure 1; Supplementary Table S1). DXS10148 was replaced by one DXSF02 in linkage group 1 (LG1) and one novel X-STR DXSF13 was added to LG3 for closer physical distance in linkage groups and accommodating more X-STRs in electrophoresis. Finally, 25 X-STRs in 7 linkage groups were adopted: LG1: DXS10135-DXS8378-DXSF02 (Xp22.31); LG2: DXSF07-DXSF08-DXSF09 (Xp21.1); LG3: DXS10079-DXSF13-DXS10074-DXS10075 (Xq12); LG4: DXSF15-DXS6803-DXSF18-DXSF19 (Xq21.31); LG5: DXSF28-DXSF29-DXSF33 (Xq23); LG6: DXS10103-DXS10102-HPRTB-DXS10101 (Xq26); LG7: DXS10146-DXS10134-DXS10147-DXS7423 (Xq28). The physical and genetic location on X Chromosome was shown in Supplementary Table S1.

All primer sets were designed using the Primer 5 software (PREMIER Biosoft, United States) and checked by the UCSC In-Silico PCR tool. The length distribution of the products was arranged from 100 to 450 bp and primers were labeled with one of four fluorescent dyes (5-FAM, HEX, TAMRA, and ROX). The detail was shown in Supplementary Table S2.

### 2.4 PCR amplification and capillary fluorescence electrophoresis

These 25 X-STR loci were amplified simultaneously in a total reaction volume of 12.5 µL containing 6.25 µL of 2× Qiagen Multiplex PCR Master Mix (Qiagen, Germany), 2 µL of primer mixture, 0.5 µL of 4 μg/μL bovine serum albumin (BSA) solution (Sangon Biotech, China), 1 µL DNA and 2.75 µL nuclease-free water according to the manufacturer’s recommendations and calibration results. PCR was performed on Mastercycler nexus GSX1 (Eppendorf, German) with the cycling condition of a temperature gradient: 5 min of initial denaturation at 94 °C; 32 cycles including 30 s of denaturation at 95 °C, 90 s of annealing at 58.5 °C and 30 s extension at 72 °C, followed by 30 min of final extension at 68 °C and stored at 4 °C. In the process of electrophoresis genotyping, 1 µL PCR product was denatured in 6 µL with a 25:1 (v/v) mixture of HiDi formamide (ThermoFisher, United States) and Size Standard Org500 (Microread, China). The mixture was then denatured at 95 °C for 4 min and cooled at 4 °C for 5 min. PCR products were separated by capillary electrophoresis on an ABI PRISM 3130xL Genetic Analyzer (Applied Biosystems, United States). Alleles of X-STRs were determined based on bins using GeneMapper ID software v3.2 (Applied Biosystems, United States) (Supplementary Figure S1). Sanger sequencing (Saiheng Biotech, China) was conducted for PCR amplification products of the new loci to confirm STR sequence structures.

### 2.5 Statistical analysis

Linkage disequilibrium (LD) analysis offers a essential method to assess the association between loci, though accurate LD estimation generally requires large-scale population datasets with phased haplotype information. This presents a challenge in forensic genetics, especially when validating newly developed loci. The maximum logarithm of the odds (MLOD) score is a commonly used statistical measure in linkage analysis, with higher scores indicating stronger evidence of linkage (Yoo and Mendell, 2008). The MLOD score was calculated with Mendel 16.0 using the Kosambi mapping function and marker allele frequencies with family data and no disease model was specified (Lange et al., 2013). and the score of >3.0 was considered significant linkage. To examine the LD characteristics among 7 linkage groups, we utilized LDBlockShow 1.4 (Dong et al., 2021) to visualize the local LD structure of 7 linkage groups based on X-SNPs from the 1000 Genomes Project. For each linkage group, a 10 kb upstream region of the first STR marker and a 10 kb downstream region of the last marker were included in the target region. (Gabriel et al., 2002). LD blocks were estimated using the Gabriel method based on pairwise D′ values. To investigate the linkage disequilibrium pattern of the X chromosome, we conducted an LD decay analysis. The analysis was performed using PopLDdecay version 3.42 (Zhang et al., 2019). We clarified that LD decay analysis was performed using pairwise *R* ^2^ values calculated across the entire X chromosome, with a MAF threshold of 0.05. The input data was extracted from the 1000 Genomes Project.

The recombination fraction of X-STRs with family data was calculated with Recombulator-X software (Aneli et al., 2023). The recombination rates calculated by phased family data of X-SNPs and the process was shown in Figure 1. We utilized the trio structure (Mother-Son or Mother-Father-Daughter) to determine the transmission of maternal X-chromosomal haplotypes. Since males inherit a single maternal X chromosome and females inherit one X chromosome from each parent, we could unambiguously trace the origin of alleles provided the mother was heterozygous. These SNPs from upstream and downstream segments of 10 kb in length close to each X-STR were used for investigation. A 10 kb flanking window was selected to align with the typical linkage disequilibrium (LD) block structure of the human genome (Gabriel et al., 2002), balancing marker informativeness with linkage specificity based on the LDdecay result. At least one heterozygotic SNP in each segment is required to be informative in determining the transmission of chromosomal segments. If both upstream and downstream segments from the same X chromosome in mother were synchronously transmitted to offspring, the chromosomal region harboring the X-STR in mother is considered to be transmitted to offspring. When two junction X-STRs in offspring originated from different X chromosomes in mother, the recombination was considered. If all SNPs within the 10 kb flanking interval were homozygous in the mother, the parental origin of the transmitted segment could not be distinguished. If the upstream and downstream flanking SNPs indicated inheritance from different maternal X chromosomes (i.e., a recombination event occurred exactly within the region spanning the X-STR), the linkage phase of the X-STR allele itself became ambiguous. To ensure accuracy, we conservatively excluded these cases rather than inferring the STR origin probabilistically. This prevents the introduction of phase errors. Informative SNPs and recombination were analyzed with in-house Python scripts and Excel treatment. The 95% confidence intervals (95% CIs) were calculated following Binomial distribution (http://statpages.org/confint.html).

## 3 Results

### 3.1 Definition and selection of 25 X-STR markers across seven linkage groups

**FIGURE 2:**
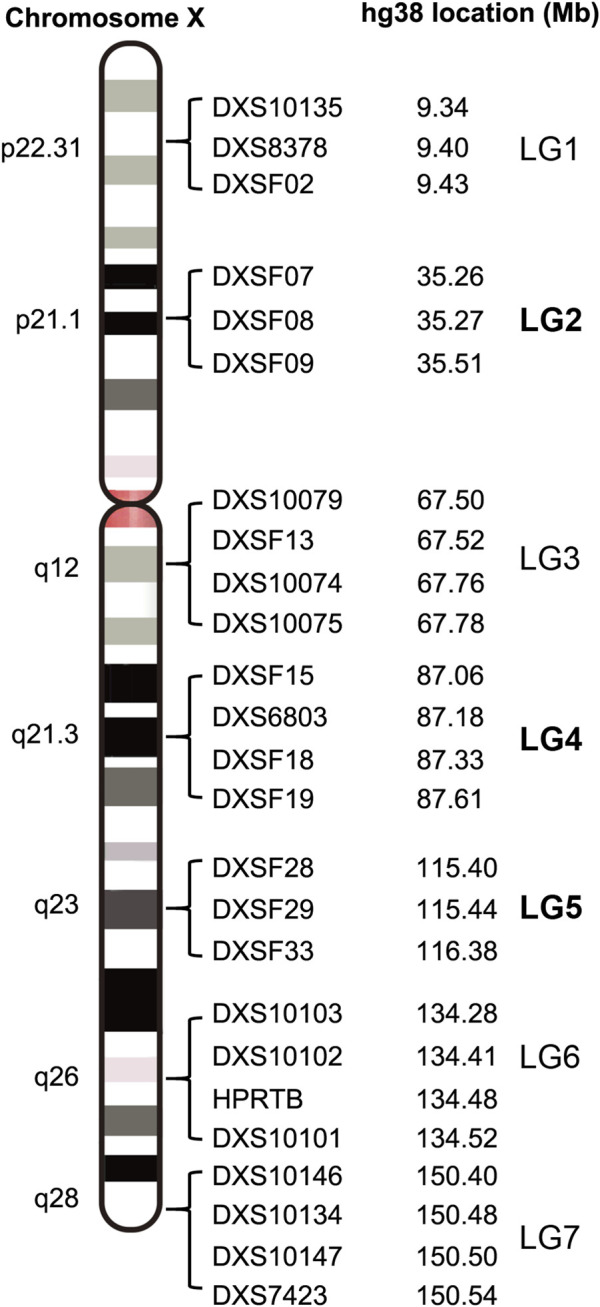
The physical location of 7 linkage groups with 25 X-STRs in X chromosome (the previously identified 3 LGs were shown in bold).

Based on the polymorphism of X-STRs on Xp21.1, Xq21.31 and Xq23 (LG2, LG4 and LG5 in this study) from our previous investigation (Yang et al., 2021) and the physical distances between X-STRs, a total of 25 X-STRs were selected for inclusion in this study (Supplementary Table S2). The physical distribution of 25 X-STRs and the location of these 7 linkage groups were illustrated in Figure 2. Following recommended nomenclature conventions (White et al., 1997; Novroski et al., 2019), the newly identified X-STR loci were named using the “DXSF0n” format. Loci previously labeled as “X0n” in our earlier study (Yang et al., 2021) have been renamed accordingly. In terms of repeat motif structure, DXSF13 consists of pentanucleotide repeats, whereas the remaining newly identified loci are composed of tetranucleotide repeat units (Supplementary Table S2). Notably, several alleles of DXSF02 contain an incomplete repeat motif. Alleles with the sequence structure of (ATAG)mATG (ATAG)n were defined as having an allele designation of (m + n).3.

### 3.2 Dual-method linkage analysis confirms within-group consistency and between-group independence

To assess linkage relationships, the MLOD values were calculated using X-STR genotypes from two-generation family datasets (Supplementary Table S3). The results showed that MLOD scores were consistently greater than 5 within each linkage group with the range of 5.0316 (DXS8378-DXSF02) to 13.8435 (DXS10146-DXS10134) and less than 1 with the range of 0.0000 (DXSF02-DXSF07, DXSF19-DXSF28 and DXS10101-DXS10146) to 0.6474 (DXS10075-DXSF15) between different groups (Table 2), indicating strong linkage within groups and independence between groups.

**TABLE 2:** The MLOD of the adjacent X-STR pairs in 7 linkage groups.

The adjacent X-STR pairs	MLOD
DXS10135-DXS8378	5.5873
DXS8378-DXSF02	5.0316
DXSF02-DXSF07	0.0000
DXSF07-DXSF08	7.1414
DXSF08-DXSF09	5.5873
DXSF09-DXS10079	0.3039
DXS10079-DXSF13	9.1719
DXSF13-DXS10074	7.6812
DXS10074-DXS10075	7.4296
DXS10075-DXSF15	0.6474
DXSF15-DXS6803	8.4265
DXS6803-DXSF18	5.0926
DXSF18-DXSF19	8.0591
DXSF19-DXSF28	0.0000
DXSF28-DXSF29	8.5702
DXSF29-DXSF33	5.8659
DXSF33-DXS10103	0.2066
DXS10103-DXS10102	8.2338
DXS10102-HPRTB	6.6835
HPRTB-DXS10101	7.7825
DXS10101-DXS10146	0.0000
DXS10146-DXS10134	13.8435
DXS10134-DXS10147	8.2488
DXS10147-DXS7423	6.4108

**FIGURE 3:**
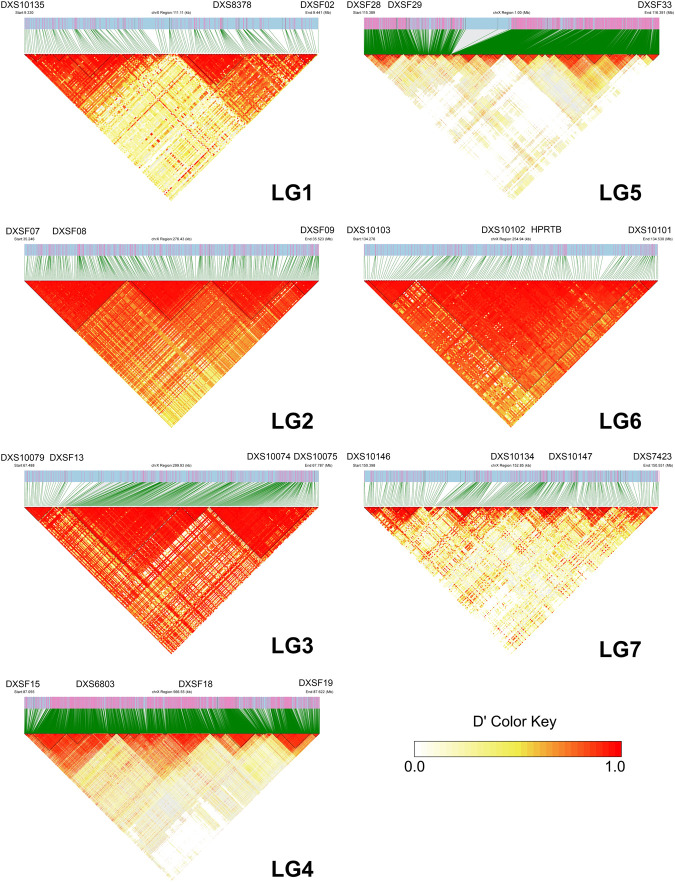
The LD heatmaps of seven X-STR linkage groups within 10 kb region included upstream and downstream. D ′value close to 1.0 indicates strong LD, while a value close to 0.0 indicates weak LD.

The LD patterns of all seven X-STR linkage groups are shown in Figure 3. Among them, LG2, LG3, and LG6 exhibited strong and continuous linkage disequilibrium across the ±10 kb flanking regions, with most SNP pairs showing complete linkage relationship. These patterns suggest stable historical co-inheritance and minimal recombination within these regions. In contrast, LG1, LG4, and LG7 displayed fragmented LD structures with multiple interspersed low-LD segments while LG5 showed particularly scattered LD segments probably due to the longest span.

To investigate the LD characteristics of the whole X chromosome, we plotted the average pairwise *R* ^2^ values of X-SNPs across different genomic distance intervals (Supplementary Table S4). The results showed that LD decayed to *R* ^2^ ≈ 0.1 at an average inter-marker distance of approximately 3.7 kb, indicating that short-range SNPs on the X chromosome still exhibit a moderate level of correlation. This pattern supports the potential of using X-SNP data to estimate X-STR recombination rates with finer resolution.

**FIGURE 4:**
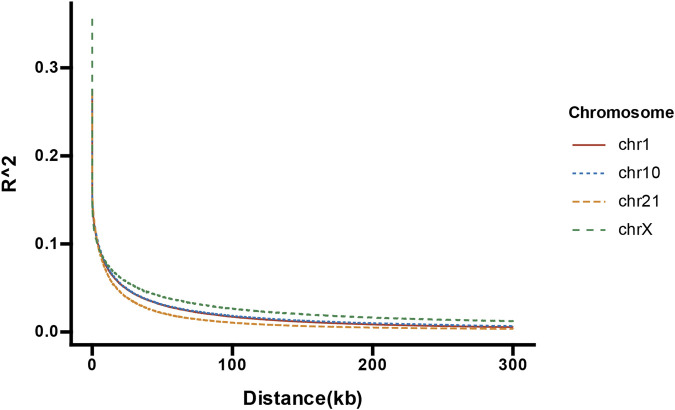
The LDdecay curve of chromosome X, 1,10 and 21.

Additionally, by comparing LD decay patterns of the X chromosome with those of representative autosomes including chromosome 1 (longest), chromosome 10 (medium-length), and chromosome 21 (shortest), we observed that the X chromosome exhibited a slower LD decay rate than all three (Figure 4; Supplementary Table S4). This distinct LD behavior of the X chromosome may reflect its unique inheritance patterns and effective population size.

### 3.3 Dual-method recombination fraction analysis confirms within-group consistency and between-group independence

**FIGURE 5:**
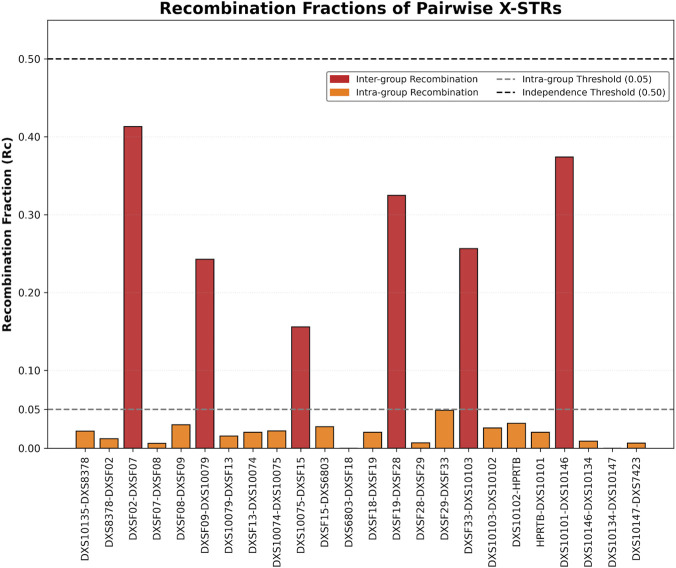
The recombination rates of pairwise X-STRs from two-generation families in this study (the intergroup recombination rates were shown in red and intra-group recombination were shown in orange).

Recombination fractions were evaluated first using the same family-based X-STR data. As illustrated in Figure 5 and detailed in Table 3, intra-group recombination rates ranged from 0.0000 (DXS10134-DXS10147 and DXS6803-DXSF18) to 0.0487 (DXSF29-DXSF33). In contrast, recombination fractions between groups ranged from 0.1561 (DXS10075-DXSF15) to 0.4133 (DXSF02-DXSF07). These results demonstrate a low frequency of recombination within clusters, whereas recombination events were relatively more frequent between clusters, supporting the classification of these seven groups as independently inherited linkage groups. The recombination fractions between common adjacent X-STR pairs generally show no significant difference compared to other studies except for the one of HPRTB-DXS10101.

**TABLE 3:** The recombination fraction between adjacent X-STR pairs using STR data and compared by previous studies.

The X-STR pairs	Rc calculated by two-generation families in this study (95%CI)	Chinese Southern Han (Yang et al., 2019)	Chinese Han (Liu et al., 2013)	Italian (Inturri et al., 2011)	Sweden (Tillmar, 2012)	German (Hering et al., 2010)	European and Asian (Nothnagel et al., 2012)	Somalis (Tomas et al., 2012)	Sri Lankan Sinhalese (Perera et al., 2022)	HapMap (Phillips et al., 2012)
DXS10135-DXS8378	0.0221 (0.0004–0.0827)	0.0000	​	0.0000	0.0000	​	0.0010	0.0000	0.1310	​
DXS8378-DXSF02	0.0125 (0.0004–0.0938)	​	​	​	​	​	​	​	​	​
DXSF02-DXSF07	0.4133 (0.2887–0.5471)	​	​	​	​	​	​	​	​	​
DXSF07-DXSF08	0.0065 (0.0000–0.0627)	​	​	​	​	​	​	​	​	​
DXSF08-DXSF09	0.0303 (0.0037–0.1065)	​	​	​	​	​	​	​	​	​
DXSF09-DXS10079	0.2429 (0.1554–0.3497)	​	​	​	​	​	​	​	​	​
DXS10079-DXSF13	0.0158 (0.0003–0.0653)	​	​	​	​	​	​	​	​	​
DXSF13-DXS10074	0.0206 (0.0028–0.0805)	​	​	​	​	​	​	​	​	​
DXS10074-DXS10075	0.0225 (0.0030–0.0862)	0.0000	0.0100	​	​	​	​	​	​	​
DXS10075-DXSF15	0.1561 (0.0858–0.2526)	​	​	​	​	​	​	​	​	​
DXSF15-DXS6803	0.0277 (0.0044–0.1252)	​	​	​	​	​	​	​	​	​
DXS6803-DXSF18	0.0000 (0.0000–0.0569)	​	​	​	​	​	​	​	​	​
DXSF18-DXSF19	0.0205 (0.0031–0.0883)	​	​	​	​	​	​	​	​	​
DXSF19-DXSF28	0.3250 (0.2224–0.4408)	​	​	​	​	​	​	​	​	​
DXSF28-DXSF29	0.0070 (0.0000–0.0507)	​	​	​	​	​	​	​	​	​
DXSF29-DXSF33	0.0487 (0.0093–0.1253)	​	​	​	​	​	​	​	​	​
DXSF33-DXS10103	0.2567 (0.1669–0.3644)	​	​	​	​	​	​	​	​	​
DXS10103-DXS10102	0.0261 (0.0027–0.0788)	​	​	​	​	​	​	​	​	​
DXS10102-HPRTB	0.0321 (0.0036–0.1034)	​	​	​	​	​	​	​	​	​
HPRTB-DXS10101	0.0206 (0.0032–0.0905)	​	​	​	0.0000	​	0.0000	0.0000	​	0.0018
DXS10101-DXS10146	0.3742 (0.2781–0.4782)	​	​	​	0.2800	0.3430	0.3220	0.3142	​	​
DXS10146-DXS10134	0.0091 (0.0003–0.0597)	​	​	​	0.0000	​	0.0205	0.0000	​	0.0017
DXS10134-DXS10147	0.0000 (0.0000–0.0425)	​	​	​	​	​	​	​	​	​
DXS10147-DXS7423	0.0068 (0.0000–0.0569)	​	​	​	​	​	​	​	​	​

Considering the limited resolution of recombination events in two-generation families, we analyzed recombination fraction with phased SNP data from 602 trios in the 1kGP (Byrska-Bishop et al., 2022). In the linkage research we observed that the flanking SNPs of certain intra-group X-STR pairs incomplete linkage and such disrupted LD patterns suggest potential historical recombination events, sparse SNP informativeness or evolutionary drift, which may influence the reliability of SNP-based recombination inference within these clusters. Given these limitations, we opted to exclude intra-group recombination estimates derived from X-SNP trio data and instead relied on pedigree-based X-STR genotypes. We restricted SNP-based inference to inter-group regions due to the scale effect. For intra-group pairs (<1 Mb), the extremely low crossover rate makes the analysis susceptible to artifacts from non-crossover gene conversion events, which can mimic crossovers in short windows (Jeffreys and May 2004; Williams et al., 2015).

The range of X-SNP searched in this study were shown in Table 4. The highest recombination fraction between X-STR linkage groups was 53.88% in LG2-LG3, while the lowest one was 45.62% in LG1-LG2. Our results showed that the recombination fraction between the 7 linkage groups occurred in approximately 50% of informative meioses (Table 5), which suggests that these 7 linkage groups should be independently transmitted in inheritance.

**TABLE 4:** The range of X-SNPs and corresponding rs ID for recombination pedigree study with 1000 Genomes Project.

X-STRs	Searched range of X-SNPs (hg38)	rs ID
DXS10135	9328425	rs778621803
	9348272	rs775485238
DXS8378	9392268	rs142655758
	9412149	rs1315888765
DXSF02	9421117	rs11795811
	9441015	rs550283087
DXSF07	35245296	rs5971932
	35259972	rs759815273
DXSF08	35265159	rs767380009
	35280046	rs73189462
DXSF09	35503675	rs142637638
	35523163	rs149509389
DXS10079	67415135	rs2077508364
	67506061	rs745563643
DXSF13	67509674	rs778054728
	67529631	rs772593647
DXS10074	67747354	rs57171883
	67767263	rs367917393
DXS10075	67768408	rs765780950
	67788346	rs779947399
DXSF15	87054170	rs756975541
	87074122	rs138592660
DXS6803	87166198	rs746146890
	87186181	rs760552881
DXSF18	87316334	rs145661595
	87336227	rs766851665
DXSF19	87601689	rs189565523
	87621551	rs747723115
DXSF28	115387331	rs187700037
	115407238	rs188963551
DXSF29	115425127	rs782745343
	115444966	rs181215721
DXSF33	116371407	rs7890707
	116391252	rs782813685
DXS10103	134275121	rs192524491
	134294757	rs150045047
DXS10102	134396132	rs769868135
	134416053	rs1035554146
HPRTB	134471543	rs761440497
	134491504	rs189207520
DXS10101	134510526	rs73560953
	134530482	rs12852452
DXS10146	150394029	rs1037584189
	150413689	rs1557402934
DXS10134	150471870	rs77285530
	150491846	rs189522806
DXS10147	150485241	rs782649114
	150505090	rs2148360591
DXS7423	150531954	rs1249404298
	150552513	rs145891900

**TABLE 5:** The recombination fraction between X-STR linkage groups using SNP data from the 1000 Genomes Project.

LG pairs	Number of observed recombination	Number of informative trios	Recombination fraction (%)	95% Confidence intervals (%)
LG1-LG2	125	274	45.62	39.62–51.72
LG2-LG3	111	206	53.88	46.82–60.83
LG3-LG4	106	226	46.90	40.25–53.63
LG4-LG5	128	263	48.67	42.48–54.89
LG5-LG6	148	296	50.00	44.16–55.84
LG6-LG7	152	285	53.33	47.36–59.24

## 4 Discussion

In this study, we investigated the linkage and recombination patterns of 25 X-STRs within seven linkage groups. Our findings, supported by MLOD analysis, recombination rates and X-SNPs investigation using the data from 1kGP, showed that these 7 linkage groups were shown to be transmitted independently and show considerable application prospects in the forensic medicine.

Although X-STRs can serve as a complementary tool for autosomal markers, the occasional recombination within clusters in allele transmission gives rise to challenges when evaluating the weight of evidence. The physical length of each cluster in this study is less than 1 Mb (genetic distance of ≤1 cM) and alleles within a cluster should be prone to linkage during transmission with a low segregation rate. Although the limitation of the sample size of family data in this study, the MLOD value of X-STR pairs and low recombination rates were observed between pairwise STR loci within a cluster in this study and our results show no significant bias compared to other family-based studies in most X-STRs in previous reports as expected (Nothnagel et al., 2012; Bini et al., 2019). The lower events of intra-group recombination are beneficial for more accurate judgment in kinship relationships (Kling et al., 2021). We also found the discrepancy for pairs like DXS10074-DXS10075 (Rc = 0.0225 despite ∼21 kb distance and complete LD) and HPRTB-DXS10101 (Rc = 0.0206 vs. prior 0.0000 (Yang et al., 2019). With the inclusion of 95% CIs, the lower bounds of observed recombination rates for pairs like DXS10074-DXS10075 approach zero. Furthermore, Fisher’s exact tests showed no statistically significant difference (p = 0.102 For DXS10074-DXS10075 and p = 0.095 For HPRTB-DXS10101 between combination rates in this study and reported by prior studies (Yang et al., 2019)). This suggests the rare events observed likely reflect stochastic fluctuations or gene conversions rather than systematic divergence. On the other hand, recombination rates may be attributed to population-specific recombination hotspots (Hinch et al., 2011), which are controlled by the highly polymorphic PRDM9 gene (Baudat et al., 2010), leading to varying recombination landscapes between Chinese Han and other populations.

The physical length of the X chromosome is 155 Mb (about 180 cM) and it is generally considered that there should be only 3 or 4 independent X-STR linkage groups on the X chromosome (Tillmar et al., 2017). However, the MLOD analysis and the investigation of X-SNPs using the 1kGP cohort both revealed an unlinked association between linkage groups during meiosis. Therefore, the inheritance of these 7 linkage groups could be considered to be independent. In contrast, a relatively low recombination rate was observed using two junction X-STRs from adjacent clusters. There might lie in the undervaluation of recombination rate due to the unphased X-STR genotypes from mothers or a small sample size (Hering et al., 2010). It might also be possible to overestimate the recombination fraction due to the occurrence of non-crossover recombination events at SNP regions (Saito and Colaiacovo, 2017). The LDdecay result of X chromosome support this: the *R* ^2^ value of LD will be reduced to 0.1 when the distance between SNP pairs is over 3.7 kb, while in X-STRs the distance is very short and almost negligible when considering linkage relationship. Non-crossover events (e.g., , gene conversion) may be detected by high-density SNP arrays as epigenetic recombination events that occur in extremely short distances, even without true chromosome fragment exchanges. If these events are mistakenly counted as restructuring, it may exaggerate the restructuring rate. However, the 10 kb window used in this study is relatively large, making it less susceptible to the effects of short distance gene switching compared to single SNP analysis (Charlesworth, 2017). The observed differences in recombination rates between family studies and population SNP data are not methodological flaws, but reflect differences in the measured biological processes (meiotic recombination and historical recombination/LD). The unique genetic pattern and effective population size of the X chromosome are key factors contributing to this paradox. It is important to note that these two strategies capture different biological timescales: pedigree analysis directly observes recent meiotic recombination events relevant to immediate kinship, whereas SNP-based LD analysis reflects historical recombination and evolutionary history accumulated over generations. Although the results of two methods have some differences, they both supported independent inheritance between studied 7 linkage groups and provide complementary perspectives and jointly depict the complex picture of X chromosome linkage relationship.

The difference of LD heatmaps between LG2-LG3-LG6 and LG1-LG4-LG7 underscores regional variation in X chromosomal linkage architecture. The contiguous high LD blocks observed for LG2, LG3 and LG6 reflect dense SNP spacing, low historical recombination and stable co-transmission, corroborating our pedigree-based evidence for tight linkage within these linkage groups. Conversely, the fragmented LD in LG1, LG4 and LG7 may arise from sparse informative SNPs, higher local recombination rates or allele frequency variation, all of which attenuate detectable LD. Conversely, the fragmented LD patterns observed in LG1, LG4, and LG7 likely reflect the presence of local recombination hotspots or structural complexities. Unlike the ‘cold’ regions of LG2 and LG6 which preserve long haplotypes, these fragmented regions may contain active recombination initiation sites defined by PRDM9 binding, leading to the rapid decay of linkage disequilibrium over short physical distances (Baudat et al., 2010). Furthermore, the X chromosome is known to harbor complex repetitive elements; such structural variations could potentially reduce the density of stable informative SNPs in these specific loci, contributing to the observed discontinuity in LD blocks (Williams et al., 2015; Song et al., 2007). These findings highlight that SNP-based linkage validation is robust for most linkage groups but also reveals structural complexity that should be considered when in forensic application. LD decay analysis further revealed that the X chromosome exhibits a slower decay rate than autosomes (e.g., chromosomes 1, 10, and 21), suggesting extended haplotype blocks. The characteristics of lower recombination rate and lower mutation rate lead to faster genetic drift. Therefore, this makes LD and population structure of the X chromosome more robust (Garcia et al., 2022). However, this does not imply stronger observable LD among X-STRs. In fact, the low recombination rate and the unique inheritance mechanism of the X chromosome (i.e., lack of male recombination) may reduce the power of LD-based analyzes in population samples. Moreover, the use of unrelated individuals may underestimate true LD due to limited ability to detect co-segregation events.

Overall, while population-based LD tests provide valuable insights into the historical genetic structure of the X chromosome, they should be viewed as complementary to, rather than a direct substitute for, pedigree analysis. Specifically, for forensic kinship applications requiring precise estimates of recent transmission probabilities, reliance solely on population LD metrics may be insufficient due to the disconnect between historical LD patterns and current meiotic recombination rate.

With the development of public WGS phased data and the database of the X-STR, future studies with deeper family structures or long-read phased sequencing may refine the linkage and recombination relationship of the X chromosome. We believe the understanding of X chromosome linkage and recombination will be more profound and accurate, and better applied in the fields of forensic science and genetics.

## Data Availability

The datasets presented in this study can be found in online repositories. The names of the repository/repositories and accession number(s) can be found below: https://www.internationalgenome.org/data, 1000 Genomes Project.
