# Supplementary figures and images for: Integrative linkage and recombination analysis of 25 X-STRs across 7 linkage groups using pedigree-based and SNP-based strategies
Source: Front Genet. 2025 Dec 18;16:1727583. doi: 10.3389/fgene.2025.1727583 (PMC12755858; doi:10.3389/fgene.2025.1727583)

**Supplementary figure**


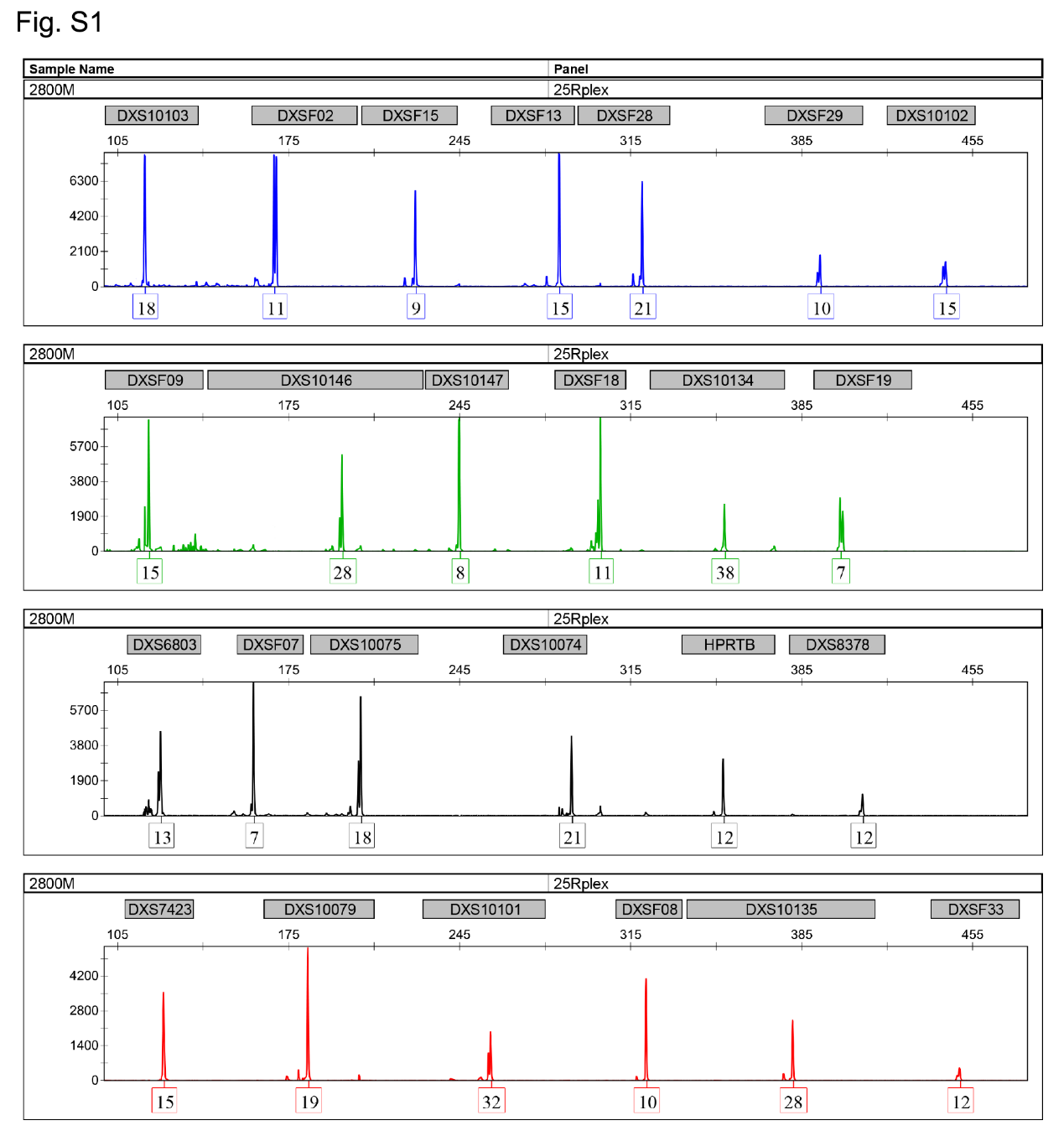


**Fig. S1** An electrophoretogram of the 25-X-STR set from 1 ng of control DNA 2800M.

Supplement: Supplementary file 2 [file DataSheet1.docx]
